# Emission of typical pollutants (NO_X_, SO_2_) in the oxygen combustion process with air in-leakages

**DOI:** 10.1007/s11356-021-14292-9

**Published:** 2021-05-09

**Authors:** Wojciech Moroń, Wiesław Ferens, Janusz Wach

**Affiliations:** Faculty of Mechanical and Power Engineering, Wroclaw University of Science and Technology, 50-370, Wroclaw, Poland

**Keywords:** Air in-leakage, Oxy-fuel, Coal, NO_x_ emission, SO_2_ emission, Devolatilisation

## Abstract

Oxygen combustion, being an alternative to air combustion, is distinguished in a variety of modern coal management technologies by quick and easy removal of CO_2_ from the combustion process, which is the key merit of this oxy-fuel technology. The laboratory work conducted so far has not directly addressed the issue of air in-leakages in the oxy-fuel system. The previous studies showed that air in-leakages in the combustion system (both under the air and oxygen regime) occur and affect the combustion process. However, there are no direct research studies on the volume of air in-leakages and their impact on the individual stages of combustion, including the emission of gaseous pollutants. This article focuses on the assessment of the impact of air in-leakages on NO_x_ and SO_2_ emissions for a single-stage coal-dust combustion system. Moreover, these studies were supplemented with measurements on the rate of devolatilisation of volatile matters and, in particular, on the rate of nitrogen compounds released from fuel. The obtained results of combustion in the oxy-fuel atmosphere with the following air in-leakage levels: 10, 15 and 20% were compared to combustion conditions in the air. Air in-leakages in the oxygen combustion system create an additional flow of oxygen and nitrogen appearing in the combustion area, which affects the course of pollutants and their emission. The conducted studies have shown that when adequate tightness of the combustion system is provided, it contributes to the reduced emission of nitrogen compounds.

## Introduction

In the early 1980s, the oxy-fuel technology with the recirculation of exhaust gases was recommended as a potential method of producing large amounts of CO_2_ for improved oil recovery (enhanced oil recovery—EOR); however, further research and development of this technology formed grounds for its implementation directly in the combustion of fossil fuels with CO_2_ separation and storage (Abraham et al. 1982; Horn and Steinberg 1982). This technology followed this path of development because it was necessary to reduce the level of CO_2_ emission to the atmosphere and reduce the negative impact of coal power engineering on the natural environment. The previous research and laboratory work focused on issues directly related to the adaptation of the oxy-fuel technology to existing blocks as well as blocks yet to be built. The details of these studies are included in numerous publications (Hill and Douglas Smoot 2000; Croiset and Thambimuthu 2001; Stanger and Wall 2011; Coraggio et al. 2011; Shaddix and Molina 2011; Shimokuri et al. 2015) and elaborations (Buhre et al. 2005; Wall et al. 2009; Zheng 2011; Baukal 2013), but they mainly focus on issues related to the analysis of the individual stages of operation within a coal unit, i.e. fuel ignition, flame stability, emission of pollutants, slagging and fouling, and heat exchange when changing the air atmosphere to O_2_/CO_2_. Also, some of the conducted work concerns issues related to the production of oxygen required for the combustion process (air separation unit—ASU). The purity of oxygen produced by an ASU is, at most, the only issue considered in the boiler oxygen-based supply system. The conducted studies indicate that when the purity level of oxygen supplied to the combustion system is lowered to 95%, it will result in a 10% reduction in the cost of its production (Wall 2007). It means that 5% of pollutants (mainly nitrogen) enter the oxy-combustion system, which in turn makes it necessary to remove additional pollutants from CO_2_ flows discharged from the combustion system. In this case, the oxy-combustion products consist mainly of carbon dioxide and water vapour together with excess oxygen required to ensure the complete combustion of the fuel. The flue gas released from the boiler will also contain other components, such as reactive and inert components derived from the fuel, e.g. SO_x_, NO_x_, fly ash, any inert components from the oxygen stream supplied (Ar, N_2_) and any inert components from the air in-leakage (N_2_, Ar, H_2_O) (Ligang Zheng 2011; Baukal 2013).

The discussed research and modelling do not take into account the amount of air in-leakages into the combustion system (Zanganeh and Shafeen 2007; Jin et al. 2015; El Sheikh et al. 2019; Li et al. 2019; Liu et al. 2019; Hu et al. 2020; Gopan et al. 2020). The modelling of the combustion and formation of NO_x_ and SO_2_ does not take into account nitrogen, which can get through leaks of the oxygen supply system or the combustion system. Conventional pulverised or fluidised bed boilers operating in the air atmosphere work with negative pressure caused by their exhaust gas fan. The fan is designed to ensure safe boiler operation by preventing the escape of exhaust gases through leaks. In a conventional combustion plant, such in-leakage represents a loss in efficiency and hence active measures are taken to keep any such infiltration to a minimum. The major sources of in-leakage air are openings in the furnace membrane wall, penetrations in the boiler casing for the hot pipework to boiler heating surfaces, at the induced draft fan, in the ash removal unit and through any gaps/penetrations/expansion joints in the ducts and casings. If it leads to difficulties in keeping the excess air ratio at the right level, problematic control of NO_x_, or keeping stable temperature, as is the case with a conventional block, then in the oxy atmosphere, the issue of leaks becomes even more relevant. Any leaks in the boiler and additional air intakes have a detrimental effect on the efficiency of the oxy-combustion process as they increase the content of nitrogen in the combustion atmosphere from one to two sources: nitrogen contained in O_2_ flows with reduced purity and nitrogen from such leaks in the combustion system. This additional source of nitrogen in the oxy combustion process can be eliminated by using the combustion process at the pressure level exceeding the atmospheric one (Lupiáñez et al. 2013; Zebian and Mitsos 2014; Liang et al. 2019).

As for SO_2_ emissions, the conducted research indicates that they are chiefly related to the content of sulphur in the fuel, while the amount of SO_2_ is proportional to the content of sulphur in coal (Carbo et al. 2009; Stanger and Wall 2011; Ligang Zheng 2011; Lupiáñez et al. 2013; Baukal 2013). The level of sulphur emissions depends mainly on the degree of fuel sulphur conversion, which in the oxy atmosphere is lower and independent of the level of in-leakages, which results in lower emissions compared to combustion in the air (Croiset and Thambimuthu 2001; Fleig et al. 2011). Air in-leakages in a conventional power plant are virtually impossible to measure and can only be estimated. Their share in newly built conventional power plants amount to about 2–4% (1–2% in a boiler and 2% in an electrostatic precipitator—ESP) (Zanganeh and Shafeen 2007; Toftegaard et al. 2010; Preusche et al. 2011; Jin et al. 2015). However, these values increase considerably during boiler operation. Some researchers claim that an increase in exhaust gas emissions can reach 10% behind the boiler, while the VGB Power Tech Report estimates that air in-leakages in power plants currently in operation range from 8 to 16% (Anheden et al. 2005; Preusche et al. 2011). The model studies presented in this paper show that, apart from the boiler, air in-leakages in the oxy-fuel system may also derive from the exhaust gas recirculation system or electrostatic precipitator, flue gas desulphurization (FGD). An increased level of air in-leakages leads to higher expenditures on the process of exhaust gas cleaning and the compression or lowering of flame temperatures (Seepana and Jayanti 2009). It should be kept in mind that, apart from oxygen which is consumed directly during the combustion process and may disturb the gradual combustion of fuel (fuel to primary/secondary oxidant) (Zanganeh and Shafeen 2007), air in-leakages are also caused by nitrogen. This gas has a fourfold higher share in the air and influences the emission of NO_x_ (Normann et al. 2009; Seepana and Jayanti 2009; Carbo et al. 2009), which consequently makes it necessary to run better purification of exhaust gases (Lupiáñez et al. 2013). In the combustion process without air in-leakages (the air in-leakage proportion: α = 0%), the maximal level of CO_2_ concentration amounts to 93.7% vol. (dry), while air in-leakages at α = 3% result in the reduction of CO_2_ concentration down to 80.9% of volume (dry). Oxygen and nitrogen are the remaining components of exhaust gases under these considerations (Preusche et al. 2011).

When fuels are combusted in oxygen, combustion products are significantly different from the ones observed when they are combusted in the air. The key difference refers to high CO_2_ and H_2_O concentration levels at the close-to-zero content of molecular nitrogen. These differences result in a significantly smaller amount of NO_x_ than in the air at similar thermal-flow conditions. At combustion in the oxy atmosphere, there are numerous possible ways to reduce nitrogen compounds (Normann et al. 2009; Toftegaard et al. 2010; Chen et al. 2012), e.g. intensified reduction of NO at char surfaces with an increase in temperature, reduction conducted in line with the Zeldovich reverse mechanism, or reduction of recirculated NO_x_ in the combustion chamber area filled with volatile matter. The conditions in the combustion area determine which mechanisms of NO formation will be predominant. Different variants of NO_x_ formation are analysed during the combustion of solid fuels cause the formation of NO_x_, e.g. thermal-NO, prompt-NO, and fuel-NO. However, in a solid fuel combustion system, the oxidation of fuel-NO is typically the most significant source of production of NO during the combustion process with some contribution from the thermal-NO. Fuel-NO emission from the oxy-fuel combustion was investigated by many researchers (Coraggio et al. 2011; Fleig et al. 2011; Shaddix and Molina 2011; Shimokuri et al. 2015), under different experimental conditions, but fuel-NO emission depended on fuel-nitrogen contents in coal. Over 80% of the NO formed in pulverised coal combustion derives from the coal, i.e. they result from the oxidation of nitrogen in the coal (coal-N), and the remainder is due to the thermal and prompt NO (Hanson and Salimian 1984; Miller and Bowman 1989; Hill and Douglas Smoot 2000; Normann et al. 2009).

The literature review shows that air in-leakages in the combustion systems, both under the air and oxygen regime, occur and have an impact on the combustion process. However, there is no research directly related to the impact of air in-leakages and their level on individual stages of combustion. This paper focuses on the assessment of the impact of air in-leakages on NO_x_ and SO_2_ emission levels for a single-stage coal-dust combustion system. The obtained results were compared to air combustion.

## Fuel samples

Two Polish coals: bituminous coal (BC) and lignite coal (LC) were used to measure pollutant emissions. The physicochemical properties of each of the fuels differ substantially and are summarised in Table 1.

**Table 1:** Proximate and ultimate analyses of fuels (on air dried basis)

Fuel		Bituminous coal (BC)	Lignite coal (LC)
Proximate analyses			
Moisture, M	%	3.1	2.0
Ash, A		8.6	17.5
Volatile matter, VM		32.7	46.6
Fixed carbon, FC		55.6	33.8
Fuel ratio, FR = FC/VM	–	1.7	0.7
Higher heating value, HHV	MJ/kg	27.7	21.7
Ultimate analyses			
Carbon, C	%	75.7	59.0
Hydrogen, H		4.3	4.8
Nitrogen, N		1.2	0.5
Sulphur, S		1.2	1.3
Oxygen (by diff.), O		5.9	14.8

The used coals are representative for the Polish coal industry and have a high higher heating value (when dry), simultaneously accompanied by a reasonable share of ash in the fuel.

## Facility and investigated cases

**Fig. 1:**
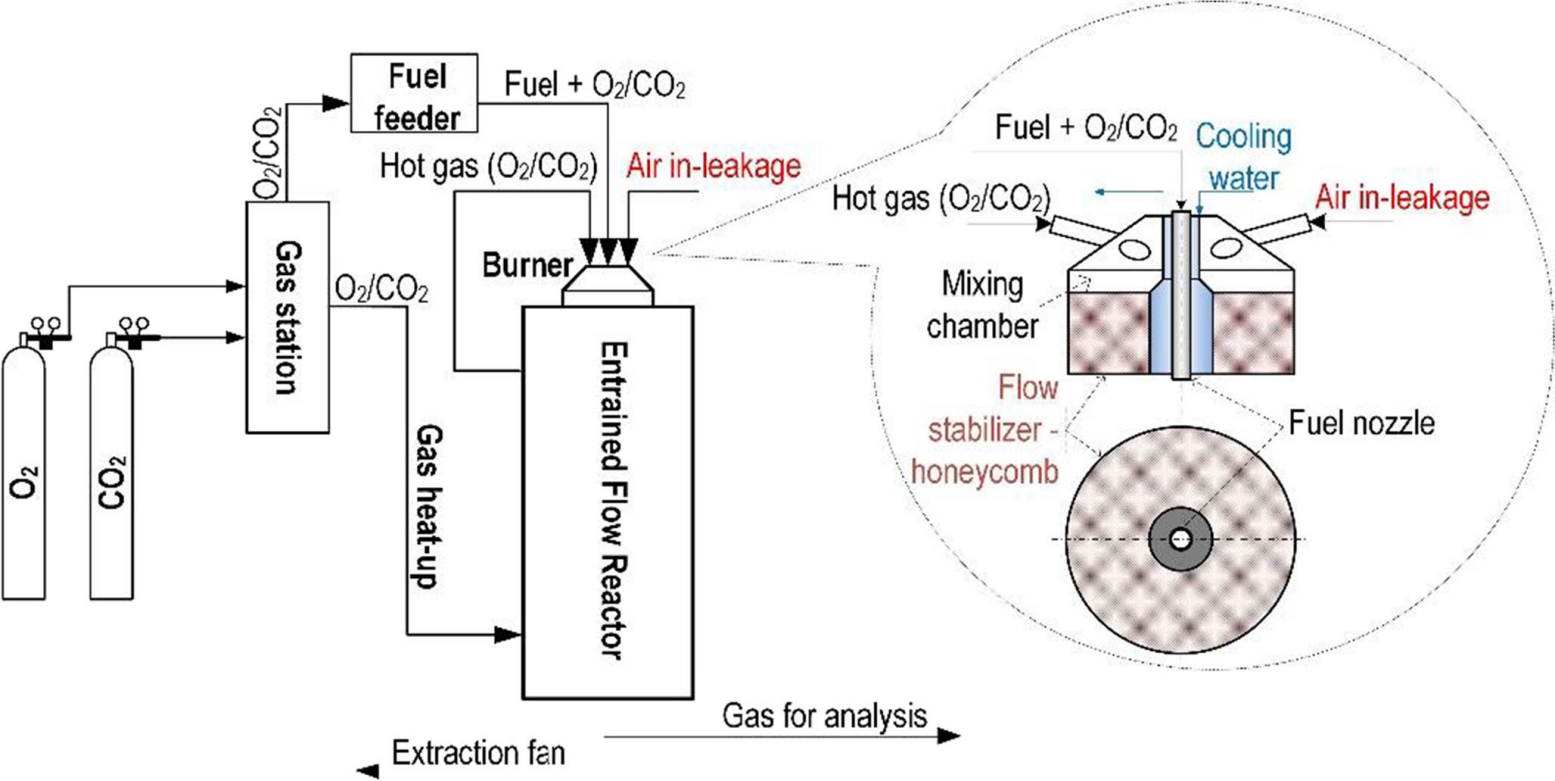
Scheme of entrained flow reactor

All the measurements were conducted in the entrained flow reactor (EFR); its diagram is presented in Fig. 1.

Briefly, the reactor reaction zone is 3000 mm long and its internal diameter is 150 mm. The reactor is electrically heated and is capable of reaching a maximum temperature of 1100 °C. The gases are preheated to the chamber temperature before being introduced into the reactor through flow straighteners. Fuel particles with a diameter under 0.2 mm were introduced through a burner with primary gas (air or O_2_/CO_2_ mixture). The concentration of the exhaust gases was measured using a spectrum analyser Siemens Ultramat 23 (O_2_, CO, CO_2_, SO_2_ and NO_x_). The experiments were performed at an elevated temperature of 1000 °C. During the fuel ignition, stable condition was kept in the furnace. The flow rate was adjusted to compensate air-leakage fluctuations and to ensure constant particle residence time in furnace. The fuel flux was in the range 5–10 g/m^3^, and the gas flux was in the range of 1–5 m^3^/h. The settings of these parameters depended on the fuel type and the type of furnace atmosphere.

**Fig. 2:**
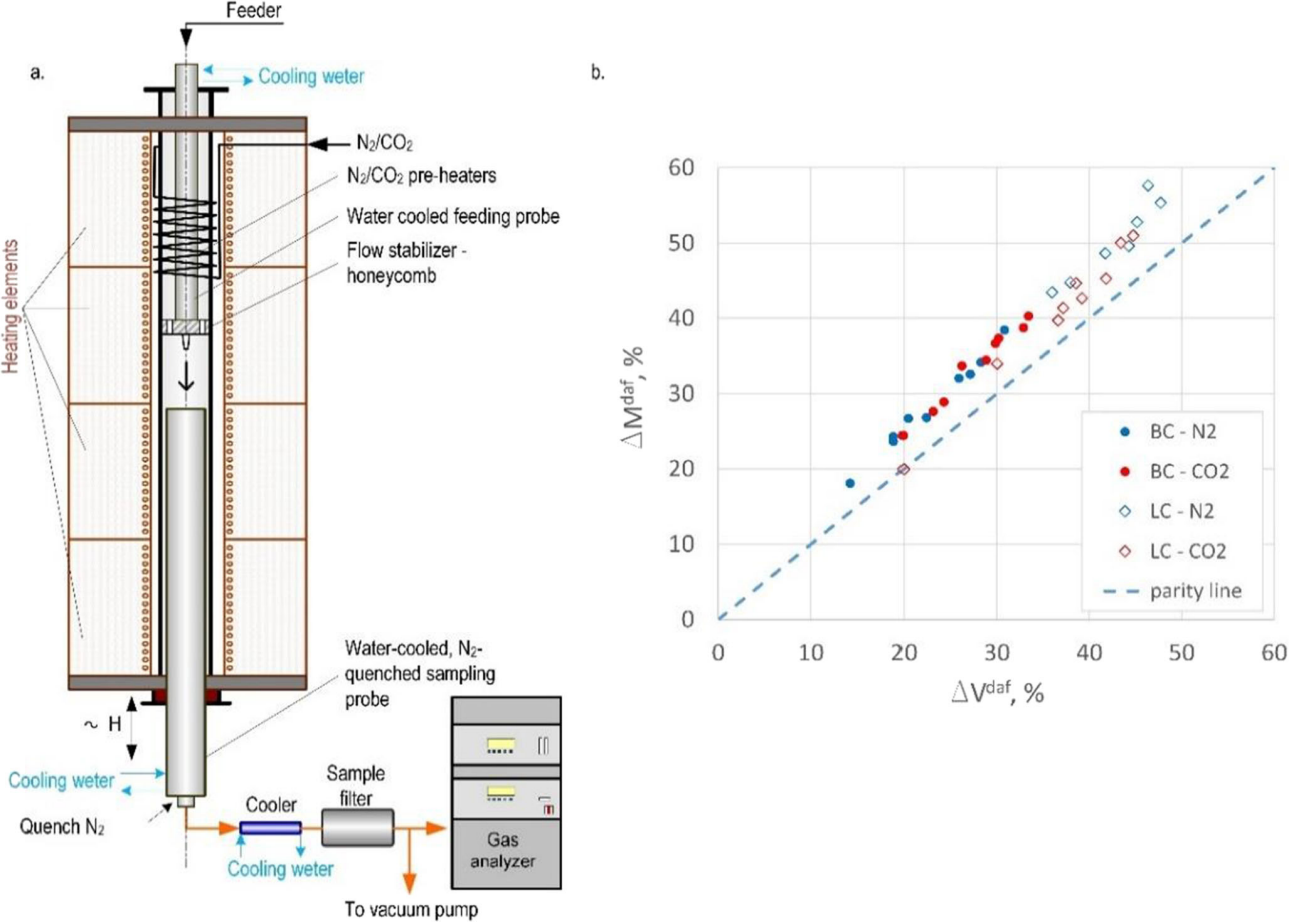
Scheme of drop tube furnace (**a**), dependence of the total content of volatile on the total mass loss (**b**)

The emission levels of NO_x_ and SO_2_ gaseous pollutants were measured at two stages. The first stage was aimed at the determination of the level of pollutant emissions for combustion conditions in the air and in the oxy atmosphere (30% O_2_/70% CO_2_). Oxy-fuel atmospheric conditions were obtained using a high-precision thermal mass flow controller (MFC) for O_2_, N_2_ and CO_2_ (Vögtlin Instruments GmbH, Red-y smart series with accuracy: ±1.0% of full scale). The second stage of the research was intended to measure the level of gaseous pollutants with air in-leakages at 10, 15 and 20% in the main gas flow. In order to imitate air in-leakages and their operation in the combustion chamber, sucked-in air was fed directly to the combustion area. The mixture of the fuel + O_2_/CO_2_ was provided as the primary flow, while air together with heated O_2_/CO_2_ (mixed) was provided peripherally as the secondary gas flow. This separation and mixing of flows guaranteed the delivery of air in-leakages directly to the primary combustion area to simulate the actual combustion system, where air in-leakages appeared in the fuel combustion chamber. Similar gas and fuel flow conditions were provided to keep similar combustion conditions (temperature distribution, residence time of fuel particles). In order to better understand the phenomena occurring during combustion in oxy-fuel conditions with air in-leakages, there were also studies on the volatile matter release and its rate in drop tube furnace (DTF) (Fig. 2a).

The stand consisted of a vertical chamber (1.5 m high) with controlled temperature and atmosphere (the control of atmospheric composition was conducted with a high-precision thermal mass flow controller (MFC) for O_2_, N_2_ and CO_2_) (Vögtlin Instruments GmbH, Red-y smart series with accuracy: ±1.0% of full scale). A uniform temperature area (1 m long) was singled out in the chamber where the temperature was measured with a K-type thermocouple in each of its heating sections. Solid and gaseous pyrolysis products were discharged from the reactor to the filtration system and then to the flue gas analyser FTIR (GASMET DX–4000). The water-cooled, N_2_-quenched sampling probe could be moved vertically, which made it possible to change the residence time of dust in the reaction area. The experiment was based on the continuous flow of pulverised fuel through an externally heated vertical reactor. Pulverised fuel and carrying gas were fed from the dosing unit through the water-cooled probe to the reactor where they were quickly heated up. The particles flowed through the reactor in short time under isothermal conditions and then were sucked in from the bottom of the reactor by a water-cooled, N_2_-quenched sample probe. In the probe, solid and gaseous products of pyrolysis were quickly cooled down. Solid products were retained in a bag filter and then subjected to the proximate and ultimate analysis. The studies were conducted for three fuel residence times within the stable area, i.e. 0.13, 0.26 and 0.40 s in three chamber temperatures: 750, 850 and 950 °C. Such measurements were made both for the devolatilisation process in the N_2_ and CO_2_ atmosphere and for the O_2_/N_2_ and O_2_/CO_2_ combustion atmosphere.

These measurements allowed to precisely determine the amount of released volatile matter, due to the fact that the amount of volatile matter which is actually released in the pulverised fuel flow ΔM^daf^ is higher than that determined by the standard analysis ΔV^daf^ carried out in the static particle bed (Fig. 2b). The fact that ΔM is higher than ΔV is due to the considerably faster (≥10^4^ K/s) heating of pulverised fuel in the flow (than in the bed) under the standard analysis (1–3 K/min). The second aspect of these measurements referred to the possibility of determining the amount of hydrogen, carbon and nitrogen compounds released in the volatile matter and the combustion of char residues, which allowed to compare the processes (devolatilisation and combustion) in the air and oxy atmosphere.

## Results and discussion

**Fig. 3:**
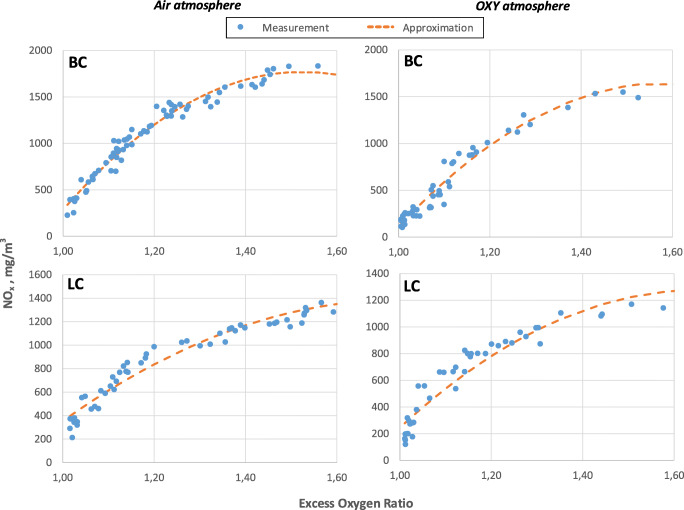
Measured emission of NO_x_ without efforts air in-leakage

**Fig. 4:**
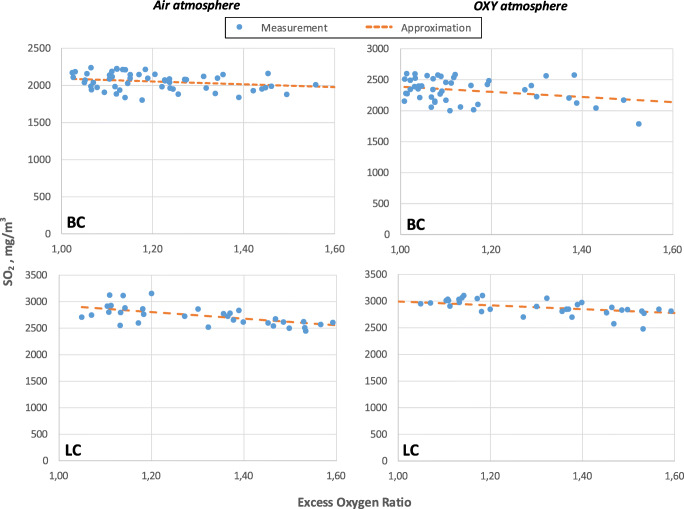
Measured emission of SO_2_ without efforts air in-leakage

The measurements were conducted for the fuels tested in the air atmosphere, oxy atmosphere (30% O_2_/70% CO_2_) and oxy atmosphere with in-leakages at 10, 15 and 20% share of air. The first step of the measurement data processing was the linearisation of the measurement curves using the least squares method. It was necessary to determine emission levels for different fuels and combustion conditions with the same concentration values of the oxidant in the chamber. The resultant measurement curves and calculation points for the fuels are presented in Fig. 3 for NO_x_ emission and Fig. 4 for SO_2_ emission. To increase excess oxygen ratio was changed only fuel flux. Increasing fuel flux not changed particle residence time in furnaces. Upon the analysis of Fig. 3 and Fig. 4, it can be stated that for SO_2_ emissions no significant variances in emissions were observed in comparison with the air and oxy atmosphere. As expected, this result is due to the fact that fuel is the only source of emission, and the previous studies and theories on the combustion process prove that the level of sulphur emission is directly proportional to its content in the fuel (Croiset and Thambimuthu 2001; Liu 2003; Stanger and Wall 2011; Chen et al. 2012). When considering the combustion system in the oxy atmosphere, it should be noted that fuel nitrogen is the key source of NO_x_ emissions (Hu et al. 2016) and the lack of nitrogen in the oxidant significantly contributes to their reduction. Taking into account the variable volume and composition of exhaust gases during combustion in the air and in the oxy atmosphere, the resultant measurement curves were converted into derivative units, i.e. mg/GJ of energy delivered to the system. The use of these derivative units eliminates the influence of the variable volume of exhaust gases in combustion in the air and oxy atmosphere (Ligang Zheng 2011; Li et al. 2015de las Obras-Loscertales et al. 2015).

**Fig. 5:**
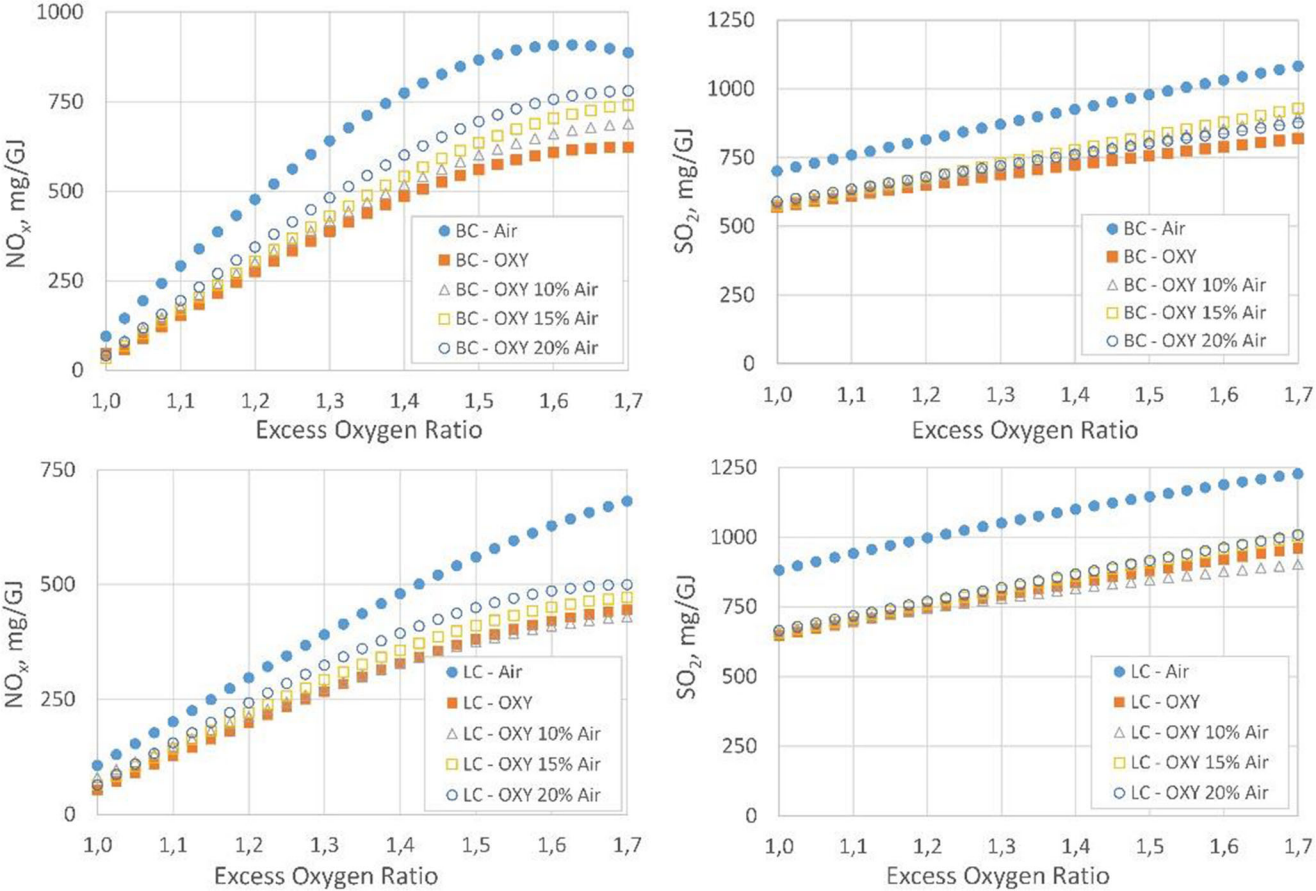
Comparison of emissions for tested fuels after conversion of units

Also, in the studies on the emission of gaseous pollutants, in the case of air in-leakages in the operational chamber, their concentration levels were recalculated (Fig. 5). This unit conversion allowed to confirm the prior observations concerning the emission of sulphur compounds. The level of the emission of sulphur compounds depends mainly on the level of fuel sulphur conversion. In the case of the oxy atmosphere, the level of fuel sulphur conversion of is about 20% lower than in the air (Fleig et al. 2011; Li et al. 2015). Moreover, the emission level of sulphur compounds is independent of the volume of in-leakages; there is no sulphur in sucked-in air, hence the emission level is kept constant regardless of the amount of in-leakages. The only discrepancies during the conducted measurements under the oxy conditions result from potential minor fluctuations in the composition of combusted fuel (averaging and sampling for falls) and the combustion process conditions.

**Fig. 6:**
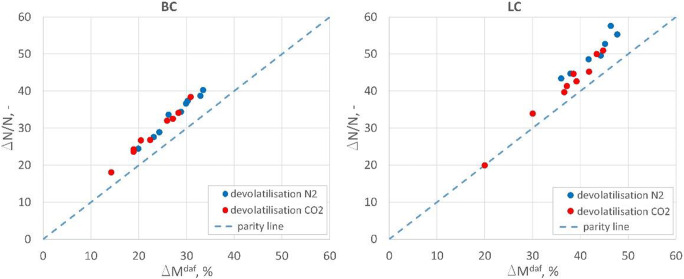
Comparison of devolatilisation of nitrogen compounds in studied atmospheres

In the case of the emission of NO_x_ nitrogen compounds, it was observed that the proportion of NO_x_ in the dust-gas flame increased along with an increase in the volume of air sucked in (Toftegaard et al. 2010). The analysis made on the volatile matter release, and in particular on the amount of released nitrogen compounds shows that in the case of the oxy atmosphere set at the level of 30% oxidant in 70% CO_2_, these processes are strongly convergent and there are no significant variances, as shown in Fig. 6.

**Fig. 7:**
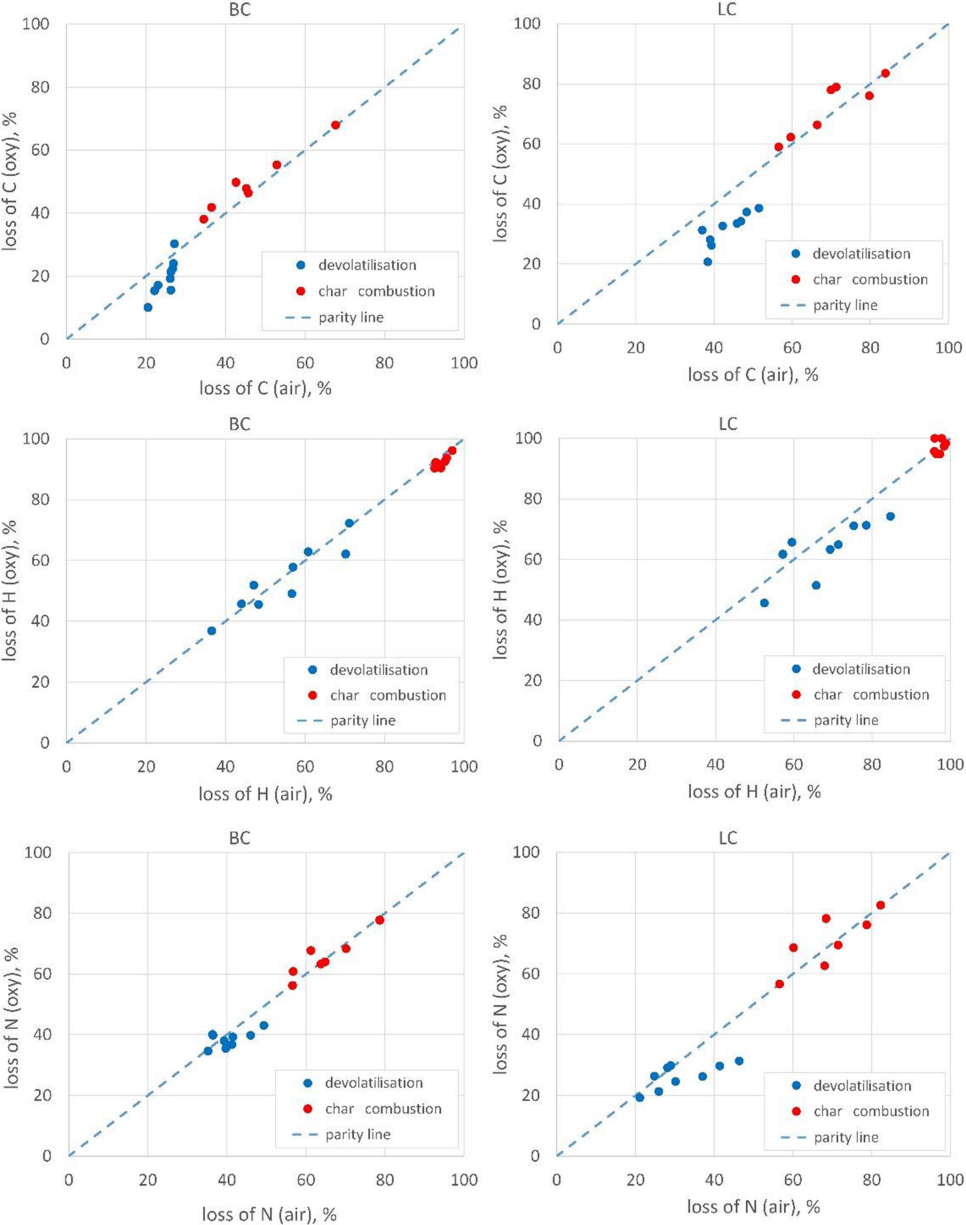
Comparison of main fuel elements: C, H and N emitted during devolatilisation and combustion process for studied atmospheres

Figure 6 shows that the relative amount of nitrogen compounds released during the volatile matter release is very similar. The variances for the tested fuels result from the coals rank: more nitrogenous compounds are released in lignite, which has more volatile matter. Also, for the tested fuels the amount of released nitrogen compounds is not proportional to the amount of released volatile matter. It should be noted that the emission of nitrogen compounds is higher than the devolatilisation rate of fuel in both atmospheres. Therefore, it can be assumed that volatile matters and reactions taking place in the gaseous phase are the main source of NO_x_ in the combustion process. For the highest degree of devolatilisation (the highest temperature and the longest residence time), it can be observed that slightly more nitrogen compounds are released in the N_2_ atmosphere than in the CO_2_ atmosphere. As for the Janina coal, it represents 5% reduction in the emission of nitrogen compounds under the devolatilisation and 21% reduction for the LC coal. The observed reduction in the release rate of nitrogen compounds during the devolatilisation process is not directly translated into reduction at the level of NO_x_ emissions under the combustion of the fuels without in-leakages in the tested atmospheres. Therefore, it should be assumed that in the studies conducted on single-stage combustion, the reduction mechanisms at the surface of char particles (Fig. 7), aside from the combustion conditions (atmospheric change), account for the reduction of NO_x_ emissions.

The analysis of the main fuel elements, i.e. C, H and N (Figure 7) shows that also in this case the combustion processes in the air and the oxy atmosphere (with the appropriate configuration of the oxy atmosphere) are very similar. For the oxy atmosphere, the amount of C and N released in the volatile matter is approximately 10% lower when compared to the one for the air atmosphere. Also, the amount of hydrogen (which may contribute to the formation of OH radicals (Eq. 1)) released together with volatile matter is slightly lower for the oxy atmosphere, which contributes to the reduction of NO_x_ emissions. The separation of nitrogen into volatile matter and char for the oxy atmosphere shows that most nitrogen is released in the combustion of char residues, which results in the reduction of NO_x_—in the presence of carbon—to molecular nitrogen.

The lack of atmospheric nitrogen in the combustion atmosphere almost eliminates other mechanisms (prompt and thermal ones) under NO formation. The distribution of fuel nitrogen between volatile matter and char is the reason why in the atmosphere, despite the increased oxygen content, local nitrogen-rich zones of higher temperature can still be formed (Figure 6). Admittedly, in these zones the prompt and thermal mechanism of nitrogen oxides formation dominates; however, the emission of nitrogen oxides in the considered combustion systems is negligible. Moreover, due to the fact that such emissions were measured making use of the artificially produced O_2_/CO_2_ atmosphere (cylinders with technical gas), the reduction mechanism of recirculation NO_x_ with fuel nitrogen and carbon-derived hydrocarbons should be excluded.

**Fig. 8:**
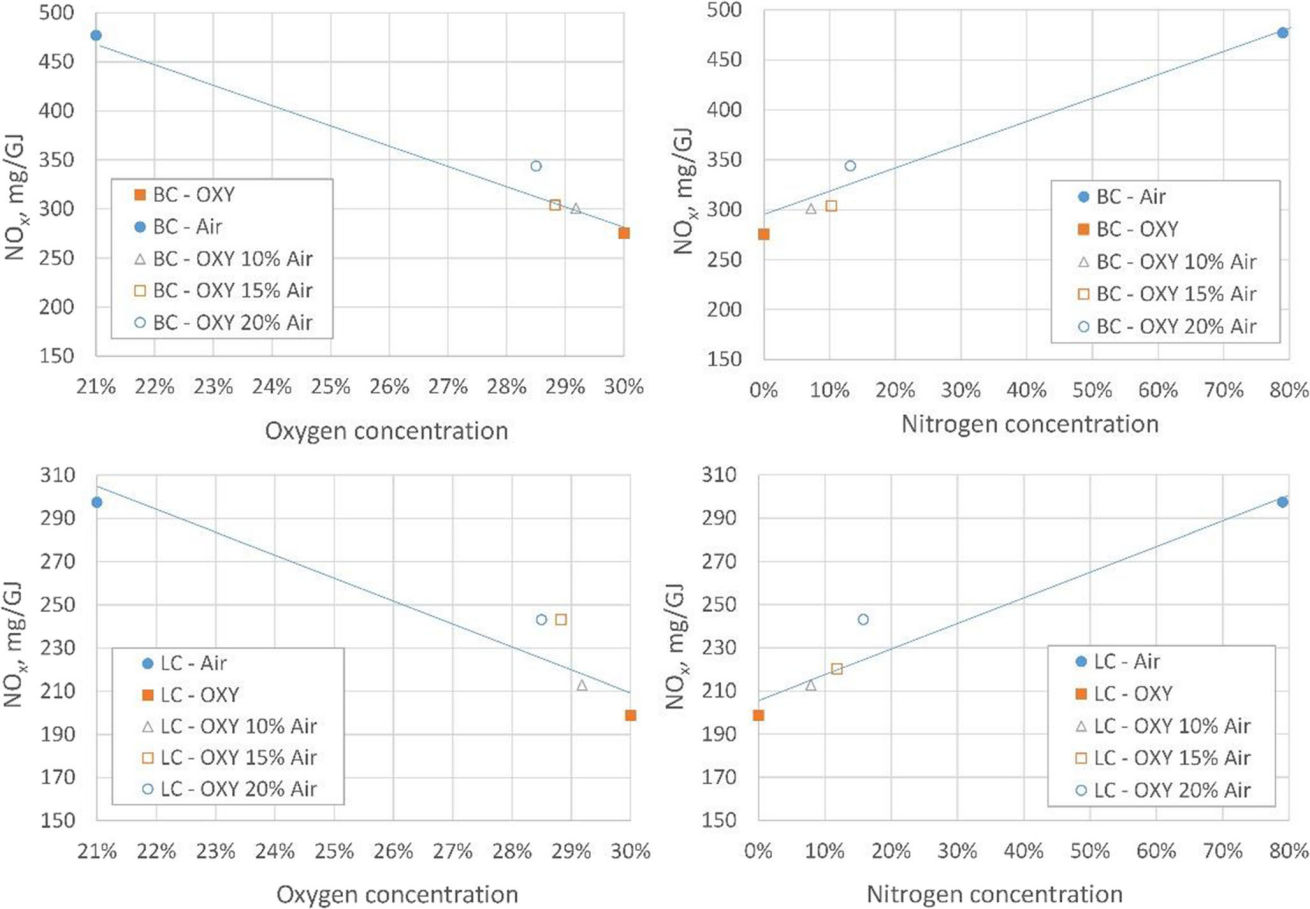
NO_x_ emission for investigated air in-leakages (for excess oxidant ratio of 1.2)

It has to be stated that air in-leakage in the combustion chamber results in the additional nitrogen in the combustion area. If it is assumed that in-leakage air consists of 21% O_2_ and 79% N_2_, then its volume will dilute the oxy atmosphere in the combustion chamber. Extra in-leakage air will lead to an increase in the volume of exhaust gases in the combustion chamber, which in turn contributes to a decrease in the amount of oxidiser in the chamber (for air in-leakage: 10, 15 and 20%, it amounts to: 29.2, 28.8 and 28.5%, respectively), a decrease in the concentration of CO_2_ (for air in-leakage: 10, 15 and 20%, it amounts to: 63.6, 60.9 and 58.3%, respectively) and the occurrence of molecular nitrogen N_2_ (for air in-leakage: 10, 15 and 20%, it amounts to: 7.2, 10.3 and 13.2%, respectively), which did not occur previously (Fig. 8). If, moreover, the oxy-fuel system is supplied with oxygen at its reduced purity (95% O_2_/5% N_2_), in the case of air in-leakage in the system at about 20% N_2_, it may cause the amount of nitrogen in the combustion area to exceed 20%. This leads to the conclusion that for the oxy-fuel technology, the control over the combustion system tightness becomes very important, and if in the case of air combustion, the level of in-leakages does not significantly affect the emission of pollutants (it mainly deteriorates the efficiency of the process), then in the case of the oxy-fuel it becomes crucial from the perspective of reducing the emission of gaseous pollutants.

The change of combustion conditions in the oxy atmosphere caused by in-leakages means that the thermal and prompt mechanisms are also responsible for the increase in NO_x_ in exhaust gases, which under the primary combustion conditions without in-leakages is excluded. It seems that in the case under consideration, the breakdown reaction N_2_ + O of molecular nitrogen (N_2_) does not take place, and this is due to the high activation energy of this reaction, which is related to high combustion temperatures (1600–1900 K), which do not occur in this case. The reactions: N + O_2_ and N + OH are relevant here (Zeldovich 1946; Hanson and Salimian 1984). Although the concentration of oxidiser in the combustion chamber decreases as a result of air in-leakage in the combustion system, it is still high when compared to the air atmosphere. Under favourable combustion conditions in the oxy atmosphere, it may result in the formation of local areas with an increased level of oxidant concentration and, consequently, lead to an increase in NO_x_ emissions by changing the combustion conditions (from reducing to oxidising).

The increased share of CO (Glarborg and Bentzen 2008; Baukal 2013) in exhaust gases during combustion in the oxy atmosphere observed during the measurements may also provide evidence on the significance of O_2_ − OH reactions. The increased concentration of CO in oxy exhaust gases is explained by the Boudouard reaction and reactions with hydrogen radicals (Eq. 1) (Glarborg and Bentzen 2008; Sutton and Fleming 2008; Torrente-Murciano et al. 2009).

1 $$ {\mathrm{CO}}_2+\mathrm{H}\to \mathrm{CO}+\mathrm{OH} $$

The second source of CO may be in the case of the CO_2_-rich atmosphere N − CO_2_ reaction (Eq. 2) (Hu et al. 2016), in addition, this reaction also generates NO.

2 $$ \mathrm{N}+{\mathrm{CO}}_2\to \mathrm{N}\mathrm{O}+\mathrm{CO} $$

The resulting OH radicals derived from the thermal decomposition of carbon dioxide (Eq. 1) in combination with the reaction N + OH and reaction products (Eq. 2) can increase the content of NO in exhaust gases.

Prompt mechanisms are complex systems of reactions, and the literature provides several suggestions on these mechanisms in which the dominant role of particular reactions or a certain group of reactions is emphasised. The Fenimore mechanism (Fenimore 1971) and the NCN-based mechanism (Miller and Bowman 1989; Sutton and Fleming 2008) are examples of such mechanisms. It seems that in the analysed case the prompt mechanism of NO formation gains significance. The formation of NO_x_ according to this mechanism takes place under the conditions of lower temperatures of fuel-rich flames, with a short residence time of fuel in the flame area. In the conducted measurements, the residence time of fuel particles in the reactor was estimated at 0.15 s and the temperature of the reactor oscillated around 1000 °C. After devolatilisation in flame, the fuel forms areas which are rich in hydrocarbons and where extra nitrogen from in-leakages appears, thus all the necessary conditions of the formation of the increased amount of NO in flames are fulfilled.

Taking into account both (thermal and prompt) mechanisms, it should be noted that as for the thermal mechanism, the resultant rate of NO formation is determined by the slowest reaction, i.e. N_2_ + O, so it should be concluded that the occurrence of extra nitrogen derived from in-leakages within the flame area will support the prompt mechanism of NO formation. The occurrence of in-leakages is not conducive to the emergence of such NO-reducing mechanisms in the oxy atmosphere. The simultaneous increase in the concentration of molecular nitrogen derived from in-leakages (disturbance of the equilibrium conditions) and the short residence time of reagents in the combustion area eliminate the reduction of NO due to the Zeldovich reversed mechanism. Also, the reduction of NO on char particles is not sufficient to offset the effect of in-leakage nitrogen on the resultant level of NO in exhaust gases. Figure 8 shows the comparison of NO_x_ emissions for the excess oxidant ratio at 1.2. It can be concluded that the increase in NO_x_ emissions in exhaust gases is proportional to the increase in nitrogen in the combustion system which enters the system as a result of in-leakages. It is also possible to observe the effect of the dilution of exhaust gases as a result of in-leakages, which causes the combustion atmosphere under the oxy conditions to tend to burn in the air if in-leakages increase further. The level of NO_x_ emissions as a function of the oxidant concentration also indicates how the operational block in the oxy-fuel technology would run during the transition from air start-up to the oxy combustion (flexi fuel).

## Conclusion

The studies conducted on the emission of pollutants under the oxygen combustion conditions and oxy conditions with in-leakages have shown quite clearly that the phenomenon of in-leakages in the oxy-fuel technology is very problematic. The previous studies did not directly consider the issue of in-leakages; this problem was acknowledged (by analogy with the process of combustion in the air) but the impact of in-leakages and their level on the emission was not analysed. The following conclusions come from the conducted research works and the analyses of the obtained measurement results:

- The processes of combustion in the oxy and air atmosphere (with the selection of the right oxy atmosphere) are very similar;
- Loss of the following elements: C, H and N in the oxy atmosphere is slightly slower than the one in the air atmosphere;
- The split of nitrogen compounds into volatile matter and char residues is very similar in the case of the two tested atmospheres, some variances result from the coals rank only;
- In-leakages in the oxygen combustion system lead to changes in the combustion conditions by disturbing the combustion atmosphere in terms of its composition, which in turn leads to the reduction in the concentration of O_2_ and CO_2_ in the chamber;
- The occurrence of in-leakages in the oxygen combustion system causes an extra flow of nitrogen to appear in the combustion area, which affects the course of the fuel combustion process. When the levels of in-leakages are high and the combustion process is based on oxygen with its reduced purity, the amount of nitrogen in the chamber may exceed 20%;
- The control over the combustion system tightness in the oxy-fuel technology is the key issue from the perspective of the reduction of the emission of pollutants with nitrogen compounds. If air enters/entered the combustion system, an increase in NO_x_ emissions is/was observed, compared to the oxy atmosphere with no in-leakages. The level of SO_2_ emissions did not alter as a result of in-leakages;
- The extra nitrogen which is present in the combustion area strengthens the thermal and prompt mechanisms of NO formation which are minimised when combusted in the oxy atmosphere without in-leakage.
